# Determination of the physiological range of oxygen tension in bone marrow monocytes using two-photon phosphorescence lifetime imaging microscopy

**DOI:** 10.1038/s41598-022-07521-9

**Published:** 2022-03-10

**Authors:** Ayako Narazaki, Reito Shimizu, Toshitada Yoshihara, Junichi Kikuta, Reiko Sakaguchi, Seiji Tobita, Yasuo Mori, Masaru Ishii, Keizo Nishikawa

**Affiliations:** 1 Graduate School of Medicine/Frontier Biosciences, Osaka University, Yamada-oka 2-2, Suita, Osaka 565-0871 Japan; 2 Laboratory of Cell Biology and Metabolic Biochemistry, Department of Medical Life Systems, Graduate School of Life and Medical Sciences, Doshisha University, Tatara Miyakodani 1-3, Kyotanabe, Kyoto 610-0394 Japan; 3 Department of Chemistry and Chemical Biology, Gunma University, Kiryu, Gunma 376-8515 Japan; 4 Laboratory of Bioimaging and Drug Discovery, National Institutes of Biomedical Innovation, Health and Nutrition, 7-6-8, Saito-Asagi, Ibaraki, Osaka 567-0085 Japan; 5 Department of Immunology and Cell Biology, WPI-Immunology Frontier Research Center, Osaka University, Yamada-oka 2-2, Suita, Osaka 565-0871 Japan; 6 Department of Synthetic Chemistry and Biological Chemistry, Graduate School of Engineering, Kyoto University, Kyoto, 615-8510 Japan; 7 WPI-Research Initiative-Institute for Integrated Cell-Material Science, Kyoto University, Kyoto, 606-8501 Japan

**Keywords:** Differentiation, Optical imaging

## Abstract

Oxygen is a key regulator of both development and homeostasis. To study the role of oxygen, a variety of in vitro and ex vivo cell and tissue models have been used in biomedical research. However, because of ambiguity surrounding the level of oxygen that cells experience in vivo, the cellular pathway related to oxygenation state and hypoxia have been inadequately studied in many of these models. Here, we devised a method to determine the oxygen tension in bone marrow monocytes using two-photon phosphorescence lifetime imaging microscopy with the cell-penetrating phosphorescent probe, BTPDM1. Phosphorescence lifetime imaging revealed the physiological level of oxygen tension in monocytes to be 5.3% in live mice exposed to normal air. When the mice inhaled hypoxic air, the level of oxygen tension in bone marrow monocytes decreased to 2.4%. By performing in vitro cell culture experiment within the physiological range of oxygen tension, hypoxia changed the molecular phenotype of monocytes, leading to enhanced the expression of CD169 and CD206, which are markers of a unique subset of macrophages in bone marrow, osteal macrophages. This current study enables the determination of the physiological range of oxygen tension in bone marrow with spatial resolution at a cellular level and application of this information on oxygen tension in vivo to in vitro assays. Quantifying oxygen tension in tissues can provide invaluable information on metabolism under physiological and pathophyisological conditions. This method will open new avenues for research on oxygen biology.

## Introduction

The bone is a highly vascularized organ with abundant blood flow^1^, but the bone marrow has been shown to be an extremely hypoxic environment^2^. As bone marrow is composed of heterogenous cell populations, including hematopoietic cells, marrow adipose tissue and supportive stromal cells^3^, it is likely that each bone marrow cell is subjected to a variety of different hypoxic environments, however, the actual oxygen levels in each cell are still not fully understood. Invasive methods to assess oxygen distribution in tissues include immunohistochemistry of pimonidazole adducts and assessment of hypoxia-inducible factor (HIF)-α accumulation^4,5^. However, these methods are neither quantitative nor indicative of oxygen tension. In contrast, real-time and quantitative measurement of oxygen tension are typically used, for example, clark electrode, where the tip of the micro-electrode is placed directly on the target tissue. However, this invasive procedure is not suitable for the measurement of oxygen tension in hard tissues, such as bone. Alternatively, non-invasive methods to quantitatively assess oxygen tension in vivo have been developed based on phosphorescence quenching^6^, electron paramagnetic resonance (EPR), and magnetic resonance techniques, including nuclear magnetic resonance (NMR) and magnetic resonance imaging (MRI)^7^. These methods have advantages and limitations in terms of applicable targets, spatial resolution, tissue permeability, convenience, and reversibility. Among these methods, optical imaging using a phosphor is advantageous for understanding the spatio-temporal dynamics of oxygen in both soft and hard tissues^8–10^. Two-photon phosphorescence lifetime microscopy imaging revealed that the extravascular oxygen tension in the bone marrow of live mice was 9.9 mmHg (1.3%) in the region 40 μm away from the bone surface and 13.5 mmHg (1.8%) in the endosteal region^10^. However, because of the use of cell-nonpenetrating phosphor, the level of dissolved oxygen in the extracellular fluid, but not the intracellular oxygen level, has been measured in vivo so far. Considering that the storage and transfer of oxygen are largely mediated by intracellular heme proteins, the intracellular oxygen levels are likely to be higher than those in the extracellular fluid, obeying Henry’s law. Indeed, using two-photon phosphorescence lifetime microscopy with a cell-penetrating phosphorescent probe that we originally developed, we found that the physiological level of oxygen in mature osteoclasts in vivo was 36.9 mmHg (4.8%), which is higher than the extravascular oxygen tension in the endosteal region previously determined^10,11^. In the present study, we found that the physiological range of oxygen tension in the bone marrow monocytes of live mice was from 2.4% to 5.3%. Furthermore, by applying information on oxygen tension in vivo to in vitro cell culture, we revealed that hypoxia in this range significantly enhanced the expression of both CD169 and CD206, which are markers of osteal macrophages.

## Results

### Physiological normoxia and hypoxia for CX_3_CR1-expressing cells in the local bone marrow environment of live mice

**Figure 1:**
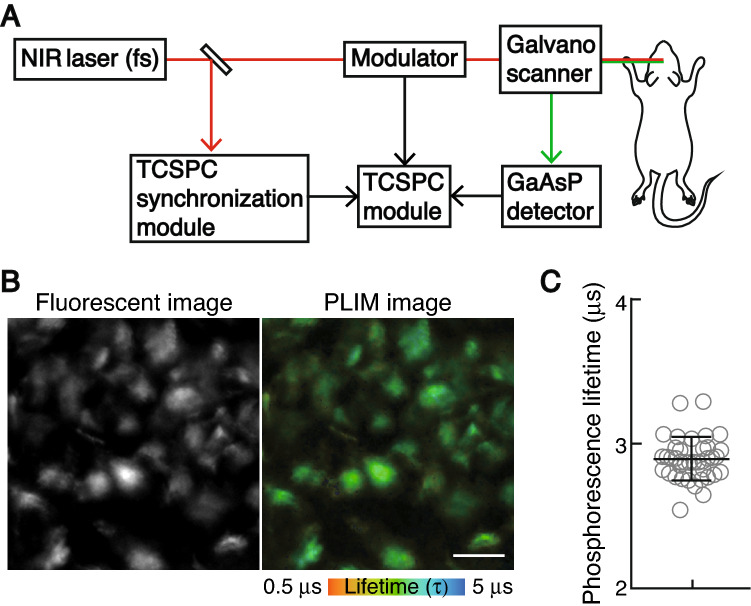
Measurement of the phosphorescence lifetime of the CX_3_CR1-expressing cells of live mice. (**A**) Two-photon phosphorescence lifetime imaging microscopy (2PLIM) setup. (**B**) Representative intravital image of the calvarial bone marrow of *CX*_*3*_*CR1*^*GFP/*+^ mice treated with BTPDM1 showing CX_3_CR1-expressing cells (left, green fluorescent protein [GFP] fluorescence) and 2PLIM image (right, phosphorescence lifetime of BTPDM1). Scale bar, 20 μm. (**C**) Phosphorescence lifetime in each CX_3_CR1-expressing cell of the calvarial bone marrow of mice upon exposure to ambient air. Data points (n = 39) represent single cells collected from four mice. Data denote the mean ± s.e.m.

A method based on the principle of phosphorescence quenching by oxygen has been successfully applied for sequential monitoring and non-invasive measurement of oxygen tension^6,12^. To measure the intracellular oxygen tension in live mice, we recently developed a method to assess the combined intensity-lifetime imaging of phosphorescence at the single-cell level, using a cell-penetrating Ir (III) phosphor (BTPDM1) and a two-photon laser scanning microscope (TPLSM) equipped with a time-correlated single photon counting (TCSPC) system (2PLIM) (Fig. 1A). To measure the phosphorescence lifetime of monocytes in the local bone marrow environment, we used *CX*_*3*_*CR1*^*GFP/*+^ mice whose monocytes express green fluorescent protein (GFP)^13^. The *CX*_*3*_*CR1*^*GFP/*+^ mice were intravenously administered BTPDM1 and subjected to intravital imaging of the calvarial bone marrow. Intravital imaging is generally performed under adequate oxygenation via the vaporization of anesthetics using medical gas, including more than 60% oxygen as the carrier gas^14^. However, in this study, to measure the oxygen tension of monocytes in the mice under physiological conditions, we used normal air as the carrier gas for the vaporizer. To maintain the mice under an adequate oxygen supply, we monitored the arterial oxygen saturation (*Sp*O_2_) using a pulse oximeter and observed that the mice exhibited *Sp*O_2_ of 97.9 ± 0.1% and heart rate of 428.9 ± 1.1 bpm (Supplementary Table 1). The GFP fluorescence enabled the visualization of monocytes, while the phosphorescence lifetime was simultaneously measured using the TCSPC system (Fig. 1B). The phosphorescence lifetime of GFP-expressing cells during air inhalation was 2.92 ± 0.03 μs (Fig. 1C).

**Figure 2:**
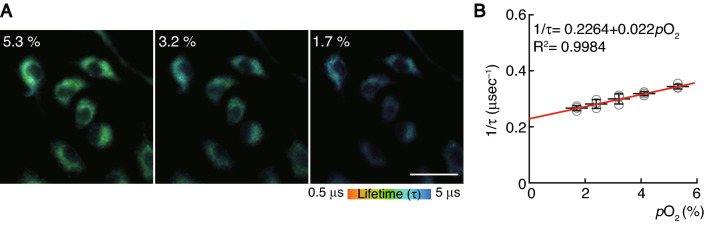
Reciprocal plot of phosphorescence lifetime and oxygen concentration. (**A**) Two-photon phosphorescence lifetime imaging microscopy (2PLIM) images of in vitro-cultured bone marrow-derived monocytes/macrophage precursor cells (BMMs) under different conditions of oxygen concentration. Scale, 20 μm. (**B**) The phosphorescence quenching due to dissolved oxygen in culture media can be examined by the Stern–Volmer equation. The approximate line was constructed by a straight-line approximation, and an approximation formula and the coefficient of determination are shown.

To calculate the oxygen tension of monocytes using phosphorescence lifetime, we prepared a calibration curve showing the relationship between phosphorescence lifetime and oxygen concentration. We cultured the bone marrow-derived monocytes/macrophage precursor cells (BMMs) treated with BTPDM1 and measured their phosphorescence lifetime under various oxygen concentrations. As expected, the phosphorescence lifetime decreased as the incubation oxygen concentrations increased (Fig. 2A). Since the decay curve of phosphorescence is described as single-exponential decay, the phosphorescence quenching due to oxygen in the culture media can be examined by the following Stern–Volmer equation:

$$ \frac{1}{{\tau_{p}^{{}} }} = \frac{1}{{\tau_{p}^{0} }} + k_{q}^{{}} pO_{2} $$

where τ_p_ is the phosphorescence lifetime, and k_q_ is the bimolecular quenching rate constant. We examined the reciprocal of the phosphorescence lifetime according to the Stern–Volmer equation. As a result, the reciprocal of the phosphorescence lifetimes fitted well with the linear approximation by oxygen concentration (Fig. 2B). Based on a calibration curve showing the relationship between the oxygen concentration and phosphorescence lifetime of BMMs, we determined that the in vivo oxygen tension of monocytes was 5.3 ± 0.2%.

**Figure 3:**
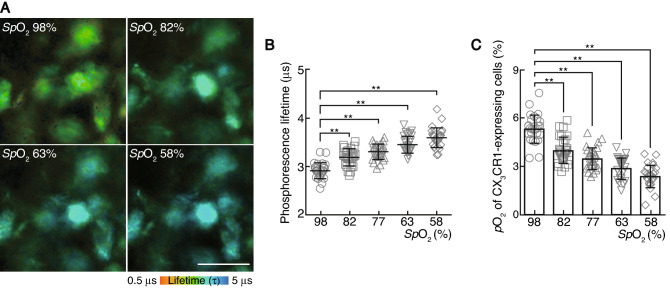
Measurement of the phosphorescence lifetime and the physiological range of oxygen tension (*p*O_2_) of CX_3_CR1-expressing cells in the mice during hypoxic air inhalation. (**A**) Change in the oxygen tension (*p*O_2_) of CX_3_CR1-expressing cells in mice upon exposure to various oxygen concentrations from 21 to 14% *p*O_2_. Magnified two-photon phosphorescence lifetime imaging microscopy (2PLIM) images of CX_3_CR1-expressing cells under different peripheral oxygen saturation values (*Sp*O_2_). Scale bar, 20 μm. (**B**) The phosphorescence lifetime of each CX_3_CR1-expressing cell in mice upon exposure to various oxygen concentration from 21 to 14% *p*O_2_ was plotted against *Sp*O_2_. Data points (n = 27) represent single cells collected from four mice. Data denote the mean ± s.e.m. **P < 0.01, one-way ANOVA with Dunnet’s multiple comparison test. (**C**) The *p*O_2_ of each CX_3_CR1-expressing cell in mice upon exposure to various oxygen concentration from 21 to 14% *p*O_2_ was plotted against *Sp*O_2_. Data points (n = 27) represent single cells collected from four mice. Data denote the mean ± s.e.m. **P < 0.01, one-way ANOVA with Dunnett’s multiple comparison test.

Next, we measured the oxygen tension of monocytes in mice exposed to hypoxic conditions to determine the physiological range of oxygen tension in monocytes. As the mice inhaled hypoxic air with 14% oxygen concentration, the *Sp*O_2_ of the mice decreased from 97.9 ± 0.1% to 58.0 ± 0.3%. When the *Sp*O_2_ fell below 58.0 ± 0.3%, the mice tended to die. Accordingly, we defined physiological hypoxia in mice as having an *Sp*O_2_ ranging from 97.9 ± 0.1% to 58.0 ± 0.3% and measured the phosphorescence lifetime of GFP-expressing cells in *CX3CR1*^*GFP/*+^ mice under several *Sp*O_2_ conditions. We found that the phosphorescence lifetime of GFP-expressing cells in these mice was prolonged to 3.19 ± 0.03 μs, 3.30 ± 0.03 μs, 3.45 ± 0.03 μs and 3.59 ± 0.03 μs at *Sp*O_2_ 81.5 ± 0.4%, 76.6 ± 0.5%, 63.1 ± 0.4% and 58.0 ± 0.3%, respectively (Fig. 3A,B, and Supplementary Table 1). Based on a calibration curve showing the relationship between the oxygen concentration and phosphorescence lifetime of BMMs, we determined that the in vivo oxygen tension of monocytes was 4.0 ± 0.2%, 3.5 ± 0.2%, 2.9 ± 0.1% and 2.4 ± 0.1% at *Sp*O_2_ 81.5 ± 0.4%, 76.6 ± 0.5%, 63.1 ± 0.4% and 58.0 ± 0.3%, respectively (Fig. 3C). These results enabled us to introduce a physiological context to oxygen tension of monocytes in vivo. Approximately 5% oxygen tension was defined as tissue normoxia (physioxia) in mice during air inhalation with an *Sp*O_2_ of 97.9 ± 0.1%; and approximately 2% oxygen tension was defined as physiological hypoxia in mice during hypoxic air inhalation with a 14% *p*O_2_, whose *Sp*O_2_ reduced to 58.0 ± 0.3%.

### Effect of physioxia perturbation on monocyte differentiation

**Figure 4:**
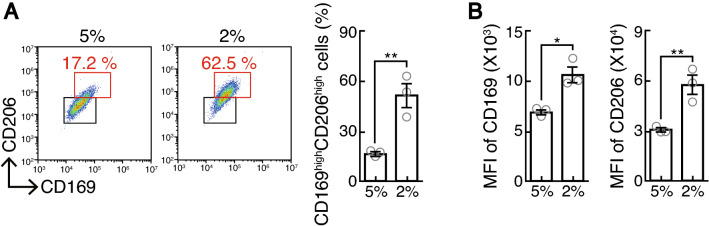
Effect of physiological hypoxia on monocyte differentiation. (**A**) Interleukin (IL)-4-treated bone marrow-derived monocytes/macrophage precursor cells (BMMs) were stained for CD115, CD169 and CD206. The CD169/CD206 profiles of CD115^+^ cells are shown. Numbers indicate the proportion of each population within the red-colored square. The percentage of CD169^+^CD206^+^ cells cultured under the conditions of 5% and 2% oxygen (n = 3, right). (**B**) Mean fluorescent intensity (MFI) of CD169 and CD206 of CD115^+^ cells (n = 3). Data denote the mean ± s.e.m. *P < 0.05; **P < 0.01, unpaired two-tailed Student’s *t* test.

Studies that have been conducted so far on the effect of hypoxia on monocyte differentiation indicate that oxygen is a negative regulator of M2 macrophage differentiation, while hypoxia promotes M1 macrophage differentiation^15,16^. We investigated the effect of physioxia perturbation on M2 macrophage polarization by applying the in vivo information on oxygen tension of monocytes to in vitro cell culture. In bone marrow, there are two types of bone resident macrophages; bone marrow macrophage and osteal macrophage. Both types of macrophages express CD169, but CD206 is expressed in osteal macrophage, but not in bone marrow macrophages^17^. We evaluated the CD169 and CD206 expression levels in vitro by culturing BMMs under the oxygen concentration of 5% and 2%. We determined that the expression levels of both CD169 and CD206 were significantly enhanced under 2% oxygen compared to those under 5% oxygen (Fig. 4A,B). These results suggest that monocyte differentiation was affected within the physiological range of oxygen tension.

## Discussion

Here, using the updated 2PLIM method, we succeeded in determining the physiological range of oxygen tension in monocytes, revealing that the precise oxygen tension exhibited by monocytes in vivo was 5.3%. Comparing the oxygen tension in the extracellular regions showing that 2.4% in the vessels and 1.3% outside the vessels in the region 40 μm away from the bone surface, and 2.9% in the vessels and 1.8% outside the vessels of the endosteal region^10^, the cellular level of oxygen tension was higher than that in the extracellular regions. This makes sense, given that oxygen is mainly stored intracellularly through the function of oxygen storage proteins, such as hemoglobin, myoglobin, cytoglobin, and neuroglobin. Thus, in addition to 2PLIM using a non-cell-penetrating phosphor, such as metal porphyrin derivatives, the previous methods including immunohistochemistry for pimonidazole adducts and micro-electrode, may underestimate the actual cellular oxygen tension in vivo.

The effect of hypoxia on cell fate decision has been studied. However, most experiments were performed under non-physiological condition, for example, by comparing a few percent below oxygen concentration as hypoxia and using atmospheric air as the control. Although the current study revealed the physiological range of oxygen tension is only a few percent, the actual impact of hypoxia in this range on cellular properties has not been well studied. Hypoxia under non-physiological condition has been previously demonstrated to promote macrophage polarization towards the M2 phenotype and increase the expression of M2 macrophage markers, such as CD206^15,16^. We demonstrated that hypoxia in the physiological range of oxygen tension leads to increased expression of both CD169 and CD206. These molecules have recently emerged as functional markers of osteal macrophage because cell depletion in mice that express diphtheria toxin receptor under the control of CD169 has been demonstrated to result in selective and striking loss of osteal macrophage^18^. The mechanism of osteal macrophage regulation is unknown, but it is possible that physioxia perturbation is involved in the differentiation of monocytes differentiation into osteal macrophages.

## Materials and methods

### Mice and bone analysis

We generated and genotyped *CX*_*3*_*CR1*^*GFP/*+^ mice as previously described^13^. All mice were born and maintained under specific pathogen-free conditions at the animal facilities of Osaka University and Doshisha University, and all animal experiments were performed in accordance with ARRIVE (Animal Research: Reporting of In Vivo Experiments) guidelines, and the Osaka University Animal Experimental Guidelines and the Doshisha University Animal Experimental Guidelines using approved protocols. The experiments were approved by the Institutional Animal Care and Use Committee of Osaka University and Doshisha University. All mice were of the C57BL/6J background.

### Two-photon phosphorescence lifetime and fluorescence lifetime imaging

Intravital microscopy of mouse calvaria bone tissues was performed as previously described^11,19^. Aged male mice (19- to 32-week-old) were used as they are typically more robust at maintaining a steady state by demonstrating a constant heart rate against surgical operation and hypoxic air inhalation, compared with female or young mice. The mice were anesthetized with isoflurane, the frontoparietal regions of the skull bones were exposed, and then the internal surfaces of bones adjacent to the bone marrow cavity were observed using two-photon excitation microscopy. The imaging system consisted of an upright two-photon microscope (FVMPE-RS, Olympus) equipped with a 25 × water-immersion objective (XLPLN 25XWMP2, N.A. 1.05; Olympus) driven by a laser (Mai Tai DeepSee, Ti:Sapphire; Spectra-Physics) tuned to 850 nm. An acoustic optic modulator (AOM) was placed in the excitation path of a two-photon microscope, enabling fast repetitive on–off switching of the laser excitation. For phosphorescence measurements, the mice were intravenously injected with 8.4 mg kg^–1^ BTPDM1^8^ before imaging. Phosphorescent and fluorescent images were collected at a depth of 100–150 μm below the skull bone surface and detected through band-pass emission filters at 525/50 nm for GFP and 620/60 nm for BTPDM1. To perform inhalation exposure of mice to low oxygen, oxygen was diluted with more than 97% nitrogen using rotameters (KOFLOC) to deliver gas mixtures containing 21% to 14% oxygen into the breathing apparatus. To monitor the degree of hypoxia in vivo, a probe attached to the thigh of the mice was used to measure the heart rate, *Sp*O_2_, and breath rate using MOUSEOX PLUS (STARR Life Sciences).

PLIM images were acquired using a DCS-120 confocal scanning system with TCSPC (Becker & Hickl GmbH/Tokyo Instruments). Single-photon emission signals were collected using emission filters (620/60 nm for BTPDM1). The SPCImage software (Becker & Hickl GmbH) was used to fit the signal of each pixel to a single-exponential decay for the phosphorescence of BTPDM1 according to the following equations:

1 $$ N ( {x,y,t} ) = A_{1} ( {x,y} )e^{{ - t/\tau_{1} ( {x,y})}} $$

where x and y are the pixel coordinates, *N* is the measured count, A is the amplitude, and t is the lifetime. Chi-square contains the squared difference between all measured data points and the modeled decay, each of which is weighted according to the expected standard deviation of the measured intensity and (for the reduced chi-square) the total number of data points:

2 $$ \chi^{2} = \left[ {\sum\limits_{i = 1}^{n} {\frac{{( {N(t_{i} ) - N_{c} (t_{i} )} )^{2} }}{{N(t_{i} )}}} } \right] \times \frac{1}{n} $$

Here, *N*(*t*_*k*_) is the measured count at time *t*_*k*_, *N*_c_(*t*_*k*_) is the modeled number of counts at time *t*_*k*_, and *n* is the number of data points. A good fit is characterized by a chi-square value close to 1 (at least, less than 1.34). We extracted both best-fit amplitudes and lifetimes from the regions of interest (ROIs) corresponding to the CX_3_CR1-expressing cells.

For in vitro analysis to generate a calibration curve of phosphorescence lifetime vs. oxygen concentration, BMMs stimulated with M-CSF for 2 days were treated with 10 μM BTPDM1, and then observed by using a two-photon excitation microscope equipped with a cell culture incubator (Stage top incubator, Tokai Hit). While diluting oxygen concentration in the culture media with more than 97% nitrogen using rotameters (KOFLOC), phosphorescent images were collected through band-pass emission filters at 620/60 nm (BTPDM1) at each concentration of oxygen in the culture media (from 5.3 to 1.7% oxygen), which was monitored using Microx 4 (TAITEC). PLIM images were acquired using a DCS-120 confocal scanning system with TCSPC (Becker & Hickl GmbH/Tokyo Instruments). Single-photon emission signals were collected using emission filters (620/60 nm). The SPCImage software (Becker & Hickl GmbH) was used to fit the signal of each pixel to a single-exponential decay according to the Eq. (1). The phosphorescence quenching due to dissolved oxygen in solution can be examined by the Stern–Volmer equation.

### Cell culture

For in vitro macrophage differentiation, bone marrow-derived cells cultured with 10 ng/ml macrophage-colony stimulating factor (M-CSF) (Miltenyi Biotec) for 3 days were used as BMMs, and were further cultured with 20 ng/ml interleukin-4 (IL-4; Biolegend) in the presence of 25 ng/ml M-CSF for 2 days. Cell cultures were exposed to physioxia (5% oxygen) and physiological hypoxia (2% oxygen) using a hypoxia workstation IN VIVO 300 (Ruskinn) and multi-gas incubator (Astec).

### Flow cytometry analysis

Flow cytometry analysis was performed as previously described^20,21^. Single-cell suspensions were incubated with anti-CD16/CD32 for 10 min and then stained with fluorescein isothiocyanate-conjugated anti-CD169 (3D6.112; BioLegend), phycoerythrin-conjugated anti-CD206 (9W9A8; BioLegend) and allophycocyanin-conjugated anti-CD115 (AFS98; BioLegend) in flow cytometry (FACS) buffer (1 × phosphate-buffered saline [PBS], 4% heat-inactivated fetal calf serum, and 2 mM EDTA) for 15 min. Stained cells were analyzed using CytoFLEX (Beckman Coulter). FACS data were statistically analyzed using the FlowJo v10.2 software (BD Life Sciences).

### Statistical analysis

All data are expressed as the mean ± s.e.m. Statistical analysis was performed using the unpaired two-tailed Student’s *t* test for comparisons between two groups and analysis of variance with the Dunnett’s multiple comparison test for comparisons among three or more groups (*P < 0.05; **P < 0.01; NS, not significant, throughout the paper)^22–24^. All data met the assumption of statistical tests and had a normal distribution, and variance was similar between groups that were statistically compared.

## Supplementary Information

Supplementary Table 1.
