# Supplementary material for: Determination of the physiological range of oxygen tension in bone marrow monocytes using two-photon phosphorescence lifetime imaging microscopy
Source: Sci Rep. 2022 Mar 10;12:3497. doi: 10.1038/s41598-022-07521-9 (PMC8913795; doi:10.1038/s41598-022-07521-9)
Supplement: Supplementary file 1 — Supplementary Table 1. [file 41598_2022_7521_MOESM1_ESM.pdf]

Supplementary Material:

Determination of the physiological range of oxygen tension in bone marrow monocytes using two-photon phosphorescence lifetime imaging microscopy

Ayako Narazaki[1], Reito Shimizu[2], Toshitada Yoshihara[3], Junichi Kikuta[1,4,5], Reiko Sakaguchi[6,7], Seiji Tobita[3], Yasuo Mori[6,7] and Masaru Ishii[1,4,5], Keizo Nishikawa[1,2,5*]

[1]*Graduate School of Medicine/Frontier Biosciences, Osaka University, Yamada-oka 2-2, Suita, Osaka 565-0871, Japan.*

[2]*Laboratory of Cell Biology and Metabolic Biochemistry, Department of Medical Life Systems, Graduate School of Life and Medical Sciences, Doshisha University, Tatara Miyakodani 1-3, Kyotanabe, Kyoto 610-0394, Japan.*

[3]*Department of Chemistry and Chemical Biology, Gunma University, Kiryu, Gunma 376-8515, Japan.*

[4]*Laboratory of Bioimaging and Drug Discovery, National Institutes of Biomedical Innovation, Health and Nutrition, 7-6-8, Saito-Asagi, Ibaraki, Osaka 567-0085, Japan.*

[5]*Department of Immunology and Cell Biology, WPI-Immunology Frontier Research Center, Osaka University, Yamada-oka 2-2, Suita, Osaka 565-0871, Japan.*

[6]*Department of Synthetic Chemistry and Biological Chemistry, Graduate School of Engineering, Kyoto University, Kyoto 615-8510, Japan.*

[7]*WPI-Research Initiative-Institute for Integrated Cell-Material Science, Kyoto University,*

Kyoto 606-8501, Japan.

*Correspondence: K.N. (kenishik@mail.dhoshisha.ac.jp)

Supplementary Table 1. Physiological parameters of the mice during air or hypoxic air inhalation.

Arterial oxygen saturation (SpO_2, %)	Heart rate (beats per minute, bpm)	Breath rate (breaths per minute, brpm)
97.9 ± 0.1	428.9 ± 1.1	79.9 ± 0.4
81.5 ± 0.4	538.9 ± 0.6	141.1 ± 0.4
76.6 ± 0.5	538.3 ± 0.5	137.3 ± 0.3
63.1 ± 0.4	525.8 ± 0.7	129.4 ± 0.2
58.0 ± 0.3	495.4 ± 0.5	116.3 ± 0.3
